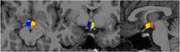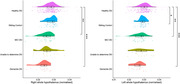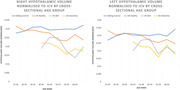# Hypothalamic involvement and biomarker candidacy in Alzheimer’s disease in Down syndrome

**DOI:** 10.1002/alz.093912

**Published:** 2025-01-09

**Authors:** Stephanie S G Brown, Shahid Zaman, John T O'Brien

**Affiliations:** ^1^ University of Cambridge, Cambridge United Kingdom; ^2^ Cambridge Intellectual and Developmental Disabilities Research Group, Department of Psychiatry, University of Cambridge, Douglas House, Cambridge United Kingdom; ^3^ Department of Psychiatry, University of Cambridge, Cambridge United Kingdom

## Abstract

**Background:**

Poor sleep is emerging as an important and modifiable risk factor in the development of dementia. The hypothalamus is the only neuroanatomical site of orexin‐producing neurones in the brain and modulates sleep and wakefulness behaviour. Due its small size and lack of defined contrast in conventional neuroimaging acquisitions, relatively little evidence exists as to the role of the hypothalamus in humans in neurodegeneration and sleep quality, and whether it may have mechanistic importance and biomarker candidacy.

**Methods:**

A total of 353 participants with DS and 37 sibling controls underwent structural brain scanning at 3T as part of the ABC‐DS international consortium research project. Hypothalami were segmented in the T1‐weighted images (Fig. 1) and assessed volumetrically as proportional to intracranial volume (ICV) and grey matter volume.

**Results:**

Whole hypothalamic volumes, when normalised to ICV, are significantly reduced in people with DS who have mild cognitive impairment (MCI) and dementia compared to those who are healthy (p < 0.05) (Fig. 2). Additionally, smaller hypothalamic volumes normalised to ICV were significantly associated with poorer cognitive performance (p < 0.05). When normalised to grey matter volume, which controls for the atrophic processes of dementia on a whole brain level, we find that healthy people with DS disproportionally smaller hypothalamic nuclei developmentally. Specifically, hypothalamic volume in the anterior‐inferior sub‐nucleus is significantly smaller in people with DS with an AD diagnosis (p < 0.05) when normalised to grey matter volume, and significantly associated with age (p < 0.05), indicating an accelerated or preferential atrophy in this location. Larger hypothalamic volumes were associated with being overweight or obese.

**Conclusions:**

These findings suggest that the whole hypothalamic structure is sensitive to AD‐related atrophic processes (Fig. 3), which are not present in sibling controls, and that hypothalamus volume is closely associated with cognitive performance in DS. The finding of an anterior‐inferior nucleus‐specific atrophy in AD which is disproportionate to whole brain grey matter loss is supportive of this region having an accelerated decline which may be linked to sleep as a risk factor or sleep‐related dementia symptomatology, as it is known to contain the suprachiasmatic nucleus.